# Supplementary material for: Identification of photoperiod-induced specific miRNAs in the adrenal glands of Sunite sheep (Ovis aries)
Source: Front Vet Sci. 2022 Jul 22;9:888207. doi: 10.3389/fvets.2022.888207 (PMC9354845; doi:10.3389/fvets.2022.888207)
Supplement: Supplementary file 1 [file Data_Sheet_1.ZIP › Supplementary file/miRDeep2 file.zip!/miRDeep2 file/Novel_Structure/LP42A3/Novel_530.pdf]

Provisional ID	:	NC_019466.2_11097
Score total	:	55.8
Score for star read(s)	:	3.9
Score for read counts	:	48.6
Score for mfe	:	1.7
Score for randfold	:	1.6
Score for cons. seed	:	
Total read count	:	107
Mature read count	:	97
Loop read count	:	0
Star read count	:	10

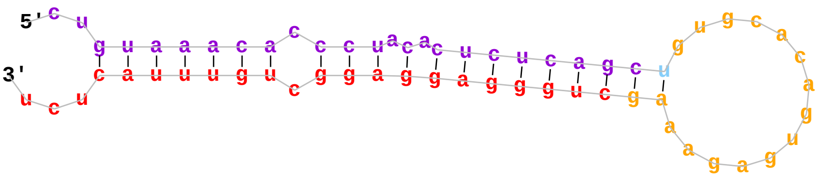

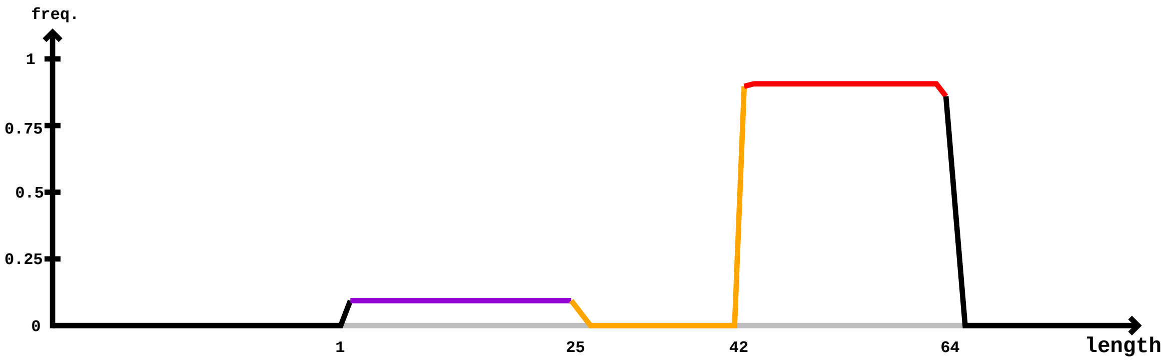


```
                            Star                                            Mature
5'- ccgccccagagagcacugagggccagauacuguaaacacccuacacucucagcugugcacagugagaaagcugggaggaggcuguuuacucucucugccuuggaagucaac   -3'    obs
    ccgccccagagagcacugagggccagauacuguaaacacccuacacucucagcugugcacagugagaaagcugggaggaggcuguuuacucucucugccuuggaagucaac          exp
    ..(((((((......))).))))((((....(((((((.(((.(.(((((((((..............))))))))))))).)))))))....))))..............   reads      mm       sample
    .............................cuguaaacacccuacacucucagc..........................................................   7          0        seq
    .............................cuguaaacacccuacacucucagcu.........................................................   3          0        seq
    ......................................................................cugggaggaggcuguuuacuc.....................   5          0        seq
    ......................................................................cugggaggaggcuguuuacucu...................   86         0        seq
    ......................................................................cugggaggaggcuguuuacucuc..................   5          0        seq
    .......................................................................ugggaggaggcuguuuacucu...................   1          0        seq
```
